# Home-Based Intervention for the Prevention and Treatment of Malaria Among Children Younger Than 5 Years in the West Region of Cameroon: Protocol for a Randomized Controlled Trial

**DOI:** 10.2196/19633

**Published:** 2021-03-12

**Authors:** Esther Dongmo Kenfack, Nicholas Tendongfor, Dickson Shey Nsagha

**Affiliations:** 1 Department of Public Health and Hygiene Faculty of Health Sciences University of Buea Buea Cameroon

**Keywords:** home-based management, malaria, children younger than 5 years, home caregivers, West Region, Cameroon

## Abstract

**Background:**

Although malaria is preventable and curable, 1 child dies of this disease every 2 minutes in Africa. Home-based management of malaria reduces the progression of severe malaria by more than 50%. Scalable, efficacious, and cost-effective strategies are needed to empower the capacities of home caregivers of children younger than 5 years of age in health education, diagnosis, and treatment of malaria at home.

**Objective:**

The main objective of this trial is to assess the impact of the management provided by home caregivers on the prevention, diagnosis, and treatment of malaria in children younger than 5 years as compared to the home-based malaria management component of the integrated community-directed intervention (CDI) strategy of community health workers (CHWs).

**Methods:**

A randomized controlled trial will be conducted. CHWs have conducted a census of all households where there is at least one child younger than 5 years with their home caregivers. These children and their home caregivers have been randomly placed into the intervention or control groups among the households identified. The trial will allow malaria home-based prevention, diagnosis, and treatment of 350 children younger than 5 years old by home caregivers in the Fombap area (intervention group) where the integrated CDI strategy will not implemented. This group will be compared to the home-based malaria management component of the integrated CDI strategy in which 350 children in the same age group will be followed up by CHWs in the Baneghang area (control group). The primary outcomes will be the prevention, diagnosis, and treatment of malaria in children younger than 5 years of age by home caregivers at home. The secondary outcomes comprise the malaria follow-up indicators produced by home caregivers in the intervention group and those produced by CHWs in the control group. Both descriptive and one-way analysis of variance estimation techniques will be used to compare the mean difference in the 2 strategies.

**Results:**

From September 2019 to October 2019, all home caregivers with children younger than 5 years of age were identified in the intervention and control group by CHWs. Following this, 203 home caregivers with their 350 children younger than 5 years were randomly selected and enrolled in the intervention group, while 225 home caregivers with their 350 children younger than 5 years were enrolled in the control group. In the intervention group, 203 home caregivers were trained in November 2019. This home treatment effectively started in December 2019 and will continue until May 2020.

**Conclusions:**

Findings from this randomized controlled trial will contribute to resolving the challenges of severe malaria and to limiting the death due to malaria of children younger than 5 years. This will bring benefits to home caregivers who will know how to promptly diagnose and properly treat malaria in their children at home.

**Trial Registration:**

Pan African Clinical Trial Registry (PACTR) 202003487018009; https://pactr.samrc.ac.za/TrialDisplay.aspx?TrialID=9788

**International Registered Report Identifier (IRRID):**

DERR1-10.2196/19633

## Introduction

About 700,000 to 2.7 million people die of malaria each year, 75% of whom are African children [1]. There were also 435,000 malaria deaths worldwide in 2017, mostly in Africa, the majority being children younger than 5 years of age, with 1 child dying every 2 minutes from this preventable and curable disease [2]. In Africa, more than 70% of malaria cases in rural areas and more than 50% of cases in urban areas are self-treated, and formal health care is sought only if initial treatment fails [3]. According to World Malaria Report 2018, similar to the top 10 most affected African countries, Cameroon recorded an increase of about 131,000 additional cases of malaria compared to the previous year [2]. The report also revealed an insufficient level of access to resources and interventions for an effective fight against malaria [2].

Most parents go to street vendors rather than to the health care system for their medications. Many studies have shown that the majority of early treatments for childhood fever are given at home [4,5] and that these treatments are usually incorrect or suboptimal [6,7].

More than 90% of sick children in rural areas in Senegal receive their first treatment at home; unfortunately, most of the time, they receive inappropriate self-medication [8]. The majority of malaria cases are recorded in households rather than in health care facilities, as they are inaccessible to the majority of those with malaria. National surveys in the World Health Organization (WHO) African Region indicate that only about one-third of febrile children receive consultation from qualified health staff [9]. Early access to effective malaria treatment is one of the main strategies for reducing the burden of malaria. This means that treatment must be available and as close to the homes as possible within 24 hours from the onset of symptoms.

Home-based management of malaria (HMM) including diagnosis by rapid diagnosis test (RDT) and treatment based on test results is a promising strategy to improve the access of remote populations to prompt and effective management of uncomplicated malaria and to decrease mortality due to malaria [10]. It has been acknowledged that it is time to intensify the HMM in endemic countries to cover the majority of their populations, and that this will result in early access to good quality antimalarial drugs at appropriate doses within 24 hours from the onset of symptoms [10]. Accumulated experience shows that it is possible to improve malaria management in the household at the community level and to reduce morbidity and mortality [11].

Studies show that HMM reduces the progression of severe malaria by more than 50% and reduces the overall mortality of children younger than 5 years old by 40% [12,13]. It is now generally accepted that with appropriate training and use of prepackaged drugs, mothers can recognize fever and administer timely and appropriate treatment [12]. In the framework of successful programs, mothers, as primary home health caregivers for their children, have been trained in the early recognition of malaria symptoms and administration of appropriate treatment [12]. The ability of mothers or home caregivers to recognize malaria and administer prompt and appropriate treatment resulted in a 40% reduction in overall mortality in children younger than 5 years old in the program area [13].

In 2001, the WHO recommended an equity strategy aimed at improving access to essential care for children younger than 5 years old through the introduction of HMM [14]. In 2010, they recommended confirmation of diagnosis by microscopy or RDT before any treatment [15]. This ensures early recognition and prompt and appropriate response (treatment) to malarial illness in children younger than 5 years within the home or the community [14]. This strategy aims to enable home caregivers to recognize malaria early and respond appropriately, to ensure that home caregivers have adequate knowledge and capacity to respond to malaria, and to create an environment that enables the strategy to be implemented by making medicines available as near to the home as possible [14]. In Senegal, a pilot study on HMM conducted in 2008 demonstrated the feasibility of integrated use of RDTs and artemisinin-based combination therapy in isolated villages by volunteer home health caregivers [10]. HMM that includes RDT and treatment based on test results is a promising strategy for improving the access of isolated populations to an early and effective management of uncomplicated malaria and for reducing malaria mortality [10]. Home caregivers have demonstrated excellent adherence to guidelines, potentially contributing to a decrease in malaria-related deaths in the community [10].

Recently, successful introduction of RDTs into HMM programs has been reported in several African countries, including Cameroon [16,17]. However, the integrated community-directed intervention (CDI) strategy has only been implemented in some health districts in Cameroon. Thus, the interventions have not been made available, particularly for children younger than 5 years; yet, this age group constitutes a vulnerable population. Innovative interventions are needed at all levels, particularly concerning prompt and effective malaria management cases, a key strategy recommended by the WHO [18].

It is very strategic to use home caregivers (mothers of children for instance) for diagnosis and administration of antimalarial treatment because they are the ones who provide first aid to their children. Community health workers (CHWs) can supplement home caregivers in their work by providing more health education, resupplying household care kits, and seeking better access to medicines. Therefore, a strategy to train home caregivers on the diagnosis and administration of malaria treatment is particularly appropriate for achieving more complete coverage. The goals of the strategy are to ensure the early recognition and prompt and effective response to malaria at home, especially for children younger than 5 years old. This strategy will not only reduce the workload of CHWs, but will also solve the unavailability and distance problem that involves people having to walk to meet the CHW for treatment. The aim of the study is to investigate the impact of a home-based intervention strategy for the prevention, diagnosis, and treatment of malaria in children younger than 5 years. We hypothesize that an intervention aimed at empowering home caregivers can make the prevention, diagnosis, and treatment of malaria in children younger than 5 years at home better than those under the integrated CDI strategy of CHWs.

## Methods

### Study Area

This study will be community-based, occurring in 2 health areas of the West Region of Cameroon: the Fombap health area in Santchou Health District where the integrated CDI strategy will not be implemented (intervention group) and the Baneghang health area in Penka-Michel Health District where the integrated CDI strategy is being implemented (control group).

**Figure 1:**
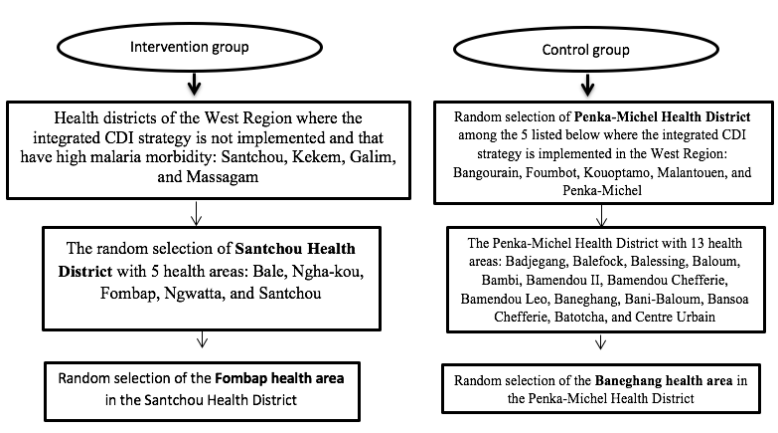
Flow chart of the sampling frame for the selection of 2 groups. CDI: community-directed intervention.

Figure 1 shows the multistage sampling approach, in which probability and nonprobability sampling methods were used to select the 2 health areas. 

### Study Design

The study will use a randomized controlled trial design. The trial was registered with the Pan African Clinical Trial Registry (202003487018009) in South Africa on March 18, 2020. It will be a community-based study examining the impact and effectiveness of the home-based malaria prevention, diagnosis, and treatment of children younger than 5 years by home caregivers (intervention) as compared to the home-based malaria management component of the integrated CDI strategy of CHWs (control). Children younger than 5 years and their home caregivers have been randomly placed into the intervention and control groups after the completion of a census of all households in the area by CHWs. In the intervention group, home caregivers will be trained to perform the RDT and administer the treatment (artesunate-amodiaquine) to children younger than 5 years for prompt and effective management at home. CHWs will be responsible for providing health education and for supplying households with malaria kits. In the control group, children younger than 5 years will be followed up through the malaria indicators produced by CHWs.

### Sample Size Calculation

The sample size calculation for this trial study will be based on the following formula for comparing 2 groups [19]:


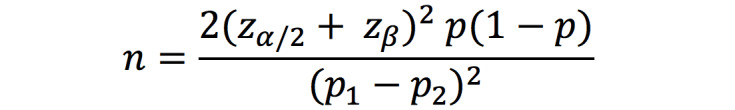


where p_1_ = 0.44, p_2_ = 0.33, Z_α/2_ = Z_0.05/2_ = 1.96 (from z table or type 1 error of 5%), Z_β/2_ = z_0.20_ = 0.842 (from z table at 80%), and p = pooled prevalence = (prevalence in intervention group (p_1_) + prevalence in control group (p_2_)/2).


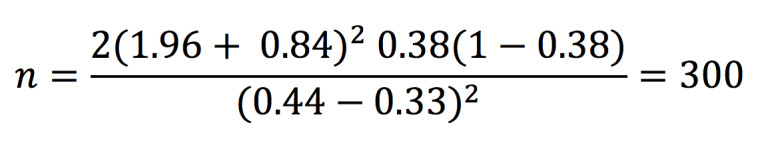


*n* = 300

With a 15% nonresponse rate, the sample size is rounded up to 350 children younger than 5 years being assigned to the intervention group and 350 being assigned to the control group.

### Inclusion and Exclusion Criteria

The inclusion criteria will be households with at least one child younger than 5 years and their home caregivers who are between 18 years and 60 years of age and who take care of these children.

The exclusion criteria will be children younger than 2 months and home caregivers who do not give their consent.

### Data Collection Procedure

Trained interviewers were blinded to group allocation and collected data using pretested questionnaires. For baseline data, a pretested questionnaire was administered to home caregivers on the perception (knowledge, attitudes, and practices) of malaria. This questionnaire will be repeated at the end of the intervention. During the intervention, a structured data collection form will be used and will contain monthly malaria follow-up indicators recorded by home caregivers in the intervention group and the malaria follow-up indicators recorded by CHWs in the control group. The data source for the study will be the malaria register used for the intervention in the Fombap health area and the malaria register used by the CHWs in the Baneghang health area.

In the experimental group (Fombap health area), 6 CHWs were chosen from the Fombap population of 5875 inhabitants, corresponding to 1 CHW per 1000 inhabitants. In each CHW’s zone, the CHW conducted a census of all the households where there is at least one child younger than 5 years and their home caregiver. From this census, children younger than 5 years old and their home caregivers were randomly selected for enrollment in the study. They will be trained in the prevention, diagnosis, and treatment of uncomplicated malaria at home. Malaria kits will be distributed to home caregivers to take care of the children younger than 5 years at home.

**Figure 2:**
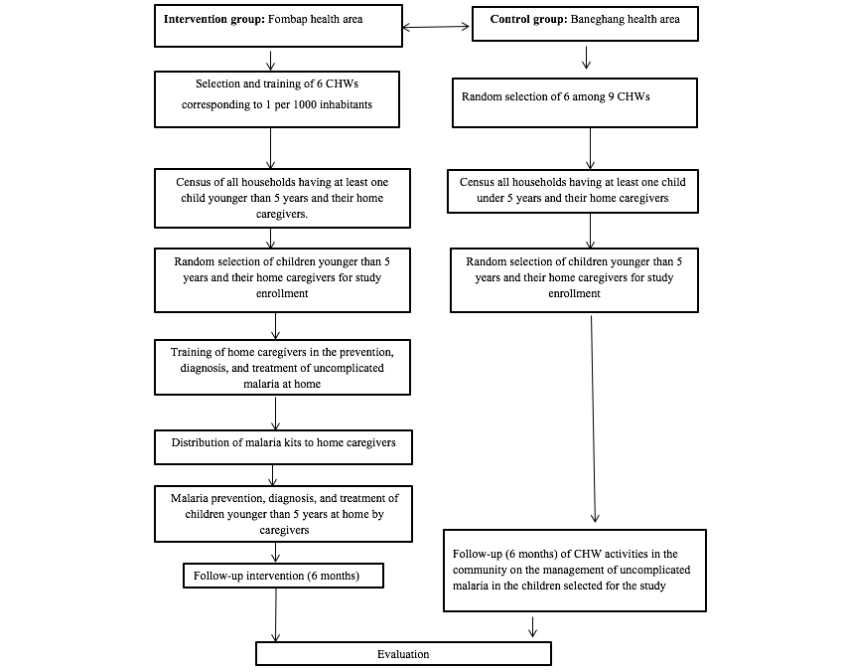
Flow chart showing the sampling method for the intervention study.

With Baneghang health area as the control group, 6 CHWs were also randomly selected from the 9 in the Baneghang health area. In each CHW’s zone, the CHW conducted a census in all the households where there is at least at least one child younger than 5 years and their home caregiver. After the households were identified, children younger than 5 years old and their home caregivers were randomly selected for enrollment in the study. Figure 2 shows the outline of the sampling method for the intervention.

### Ethical Considerations

Ethical approval has been obtained from the Faculty of Health Sciences Institutional Review Board of the Buea University (reference no. 2019/1023-09/UB/SG/IRB/FHS). Administrative authorization has been obtained from the University of Buea, the Regional Delegation of Public Health for the West Region, and the health districts of Santchou and Penka-Michel.

Participation of individuals and households will be strictly voluntary and on the basis of informed written consent and assent by the household heads or caregivers. The purpose of the study and the role of the participants will be well explained in the consent form. Once informed consent and assent are obtained from the participants, research staff will collect baseline data, and will conduct the intervention and postintervention.

### Intervention Development

In order to contribute to the improvement of the HMM component of the integrated CDI strategy, an intervention will be implemented to make home caregivers accountable for the malaria prevention, diagnosis, and treatment at home. This strategy will be used in the Fombap health area where the integrated CDI strategy will not be implemented. The intervention was designed with the active participation of CHWs and home caregivers in the management of malaria at home through their training. Participants in this study will be made aware that the training they receive will give them the opportunity to test and treat malaria in their children younger than 5 years of age at home. Thus, this intervention will enhance home caregivers’ capacities to prevent, diagnose, and treat malaria at home in their children younger than 5 years. The different steps will be the selection of CHWs, the census of households with at least one child younger than 5 years and their home caregivers, the training of CHWs and home caregivers, the provision and the management of malaria kits, the health education of home caregivers on malaria prevention measures, and the monitoring and evaluation of the activities.

#### Step 1: The Selection of Community Health Workers

In the intervention group, the identification and selection of CHWs will be carried out in collaboration with the head of the health area and the leaders of all communities. A meeting will be convened at the Fombap Higher Chiefdom during which CHWs in each community will be selected. Then, 6 CHWs will be selected in the intervention group and 6 CHWs will be randomly selected among the 9 existing in the control group.

#### Step 2: The Census of Households, Children Younger Than 5 Years, and Home Caregivers

The CHWs in their respective zones identified and recorded all households with at least one child younger than 5 years and their home caregivers. After identifying the households, the children younger than 5 years and their home caregivers were randomly selected by the researchers for enrollment in the study.

#### Step 3: The Training of CHWs and Home Caregivers

**Figure 3:**
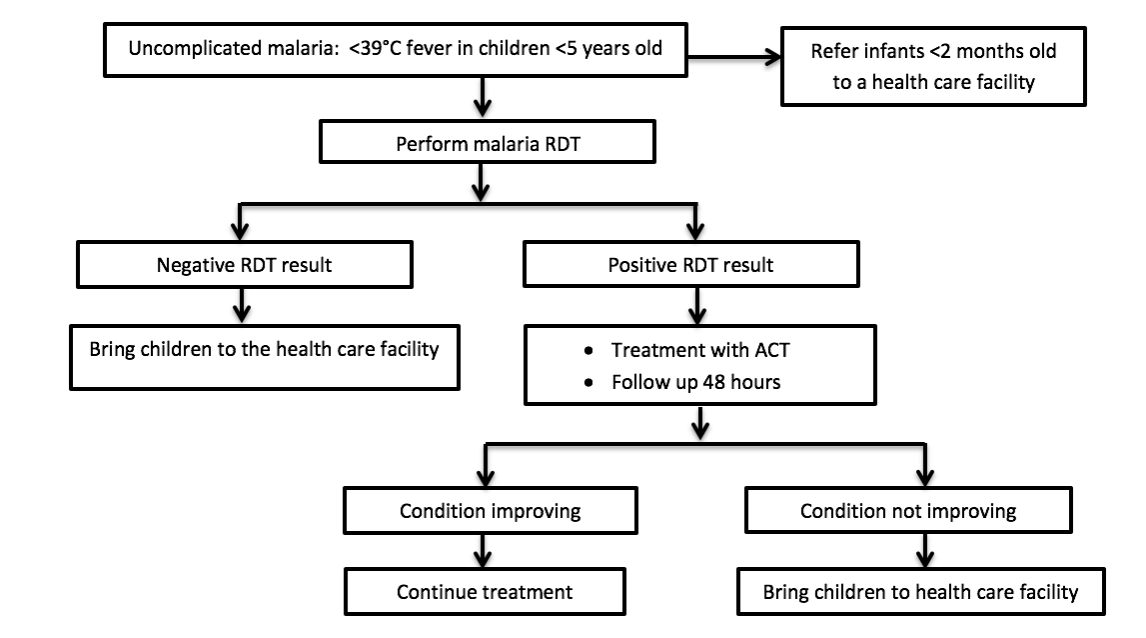
Algorithm for the home management of malaria adapted from that used in Senegal. ACT: artemisinin-based combination therapy; RDT: rapid diagnosis test.

The selected CHWs will be trained by the research team, and home caregivers will be trained by the research team with the help of the CHWs. The training will be focused on malaria management of children younger than 5 years at home and will include recognizing uncomplicated malaria (base on sign and symptoms), performing malaria RDT, administering artesunate-amodiaquine to children in the case of a positive test, and referring RDT-negative children or those with severe malaria to the health care center. The training process will be based on the training handbook of the Cameroon Ministry of Public Health. The training module of the CHW modified version will be used, as it is simple and has practical demonstrations to enhance understanding. The algorithm used in our study (Figure 3) will be adapted from that used in Senegal [10].

#### Step 4: The Provision and Management of Malaria Kits

SD Bioline Malaria Ag Pf RDT is used in Cameroon and meets the performance criteria recommended by the WHO [20], while a fixed dose of artesunate-amodiaquine is a combination used as first-line treatment in Cameroon [21]. RDTs and malaria kits will be distributed during the training to all home caregivers, and then the CHWs will take care of replacing kits in households. The malaria kits will be provided to CHWs by the research supervisor according to the need expressed. The support kit will consist of a thermometer, 2 RDTs, a packet of 25 mg/67.5 mg of artesunate-amodiaquine and/or 50 mg/135 mg of artesunate-amodiaquine, a packet of paracetamol, and an algorithm chart for malaria management. In case of any fever above 38ºC in the children younger than 5 years of age as confirmed by the thermometer and without any other sign of gravity, the home caregiver will conduct an RDT of malaria. If the test result is positive, the caregiver will administer the child artesunate-amodiaquine and paracetamol; if the result of the test is negative, the caregiver will take the child to a health center for in-depth exams and treatment for the other causes of fever. For statistical purposes and to replace their kits, home caregivers will inform the CHW of their community by phone of the results of the test and the treatment received by their child. Malaria kits will be distributed free of charge for children younger than 5 years old and this will be purchased from the national provision fund. This study is funded by EDK.

#### Step 5: The Health Education of Home Caregivers

CHWs will provide health education to the home caregivers concerning malaria prevention measures, the advantages of HMM, and the role of home caregivers in the health care of children. Health education will focus on malaria prevention measures, such as cleaning around the house, the use of insecticide-treated mosquito nets, the use of window screens, the use of insecticides, and the closure of doors and windows before nightfall. CHWs will also explain the concept of home-based prevention and treatment of malaria and the possibility of treating uncomplicated malaria in the community and even in the home. They will also explain that if the diagnosis and treatment of uncomplicated malaria occurs within 24 hours, the condition usually progresses to cure, whereas if early diagnosis and treatment do not occur, the condition can progress to severe malaria and even death. CHWs will provide health education to home caregivers by going door-to-door once a week to at least 5 households and will also go once a month to meetings of women in their area. They will also go to replace the malaria kits in any household that has a suspected case.

#### Step 6: Monitoring and Evaluation Activities

The monitoring of the activities will be carried out by the research supervisor, the head of the health area, and the researchers to compile and evaluate the work completed on the field. A meeting will be organized twice a month, bringing together the research supervisor and the CHWs to monitor activities. For statistical purposes and to replace their kits, home caregivers will inform CHWs in their community of all suspected malaria cases in the children younger than 5 years, of the result of RDTs, and of the malaria treatment received by the child. Each month, the monitoring indicators produced by the home caregivers will be collected by CHWs for evaluation purposes. An evaluation of the activities will be carried out monthly during the period of intervention.

After the intervention, a descriptive, analytical, and comparative study will be made in the Baneghang and Fombap health areas to compare the two strategies. This evaluation will be based on the HMM follow-up indicators reported by home caregivers in the intervention group and will be compared to those of the CHWs in the control group.

### The Control Group

The integrated CDI strategy is implemented in 5 health districts in the West Cameroon region, including the Penka-Michel Health District, from which our control health area, Baneghang, was randomly selected for the site of the control group. In this area, the malaria management component of the integrated CDI strategy is carried out by 9 CHWs, 6 of whom were randomly selected for the study. CHWs conducted a census in their respective zone of all the households where there is at least one child younger than 5 years and their home caregivers. After identifying the households, children younger than 5 years old and their home caregivers were randomly selected for enrollment in the study.

Usually, when there is a suspected case of malaria in the community, the patient is taken to the CHW's home. The patient is examined by the CHW to check for uncomplicated malaria. If the test is positive, the CHW conducts an RDT and administers malaria treatment; if the test is negative, the CHW refers these patients and those with severe malaria to the health care center and makes home visits to provide health education to parents.

### Presentation of the Two Strategies

**Figure 4:**
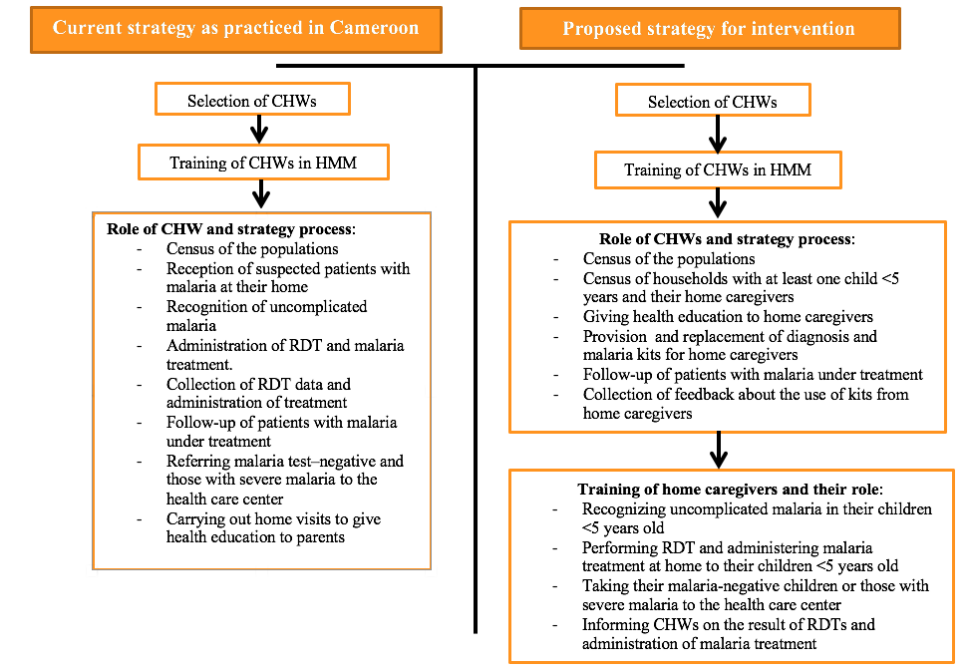
Flow chart of the two strategies. CHW: community health worker; HMM: home-based management of malaria; RDT: rapid diagnosis test.

Figure 4 shows the current strategy put in place by the Ministry of Public Health through the integrated CDI and intervention approaches.

### Outcome Measures

#### Primary Outcomes

The primary outcomes will be the prevention, diagnosis, and treatment of malaria in children younger than 5 years of age by home caregivers at home as measured by comparing malaria follow-up indicators produced by home caregivers in the intervention area to those produced by CHWs in the control group.

#### Secondary Outcomes

The secondary outcomes will be the proportions of malaria follow-up indicators, including the proportion of suspected malaria cases, the proportion of confirmed malaria cases, the proportion of confirmed malaria cases who have received artesunate-amodiaquine, the proportion of children younger than 5 years referred to and who received treatment in health care facilities in the health area, and the proportion of household visits made by CHWs.

### Data Management and Analysis

Data management and statistical analysis conducted during the intervention will be entered into the research log book and checked by a statistician. All data will be encoded and accessible only to those allowed by EDK The local ethics committee has already approved the study and confirmed the detailed specifications on data security provided to them. The data will be later keyed into a computer using Microsoft Excel 2016 (Microsoft Corporation) and checked for input errors. Descriptive analyses will be conducted using mean, SD, median, IQR, and counts, based on distribution. To investigate the effects of the intervention, a comparison of the pre- and postintervention data will be conducted using the Wilcoxon signed-rank test for the indicators of the malaria. For group comparisons, the *t* test will be used to compare means, while the chi-square test will be used to compare the proportions of malaria home-based management indicators in the intervention and control groups. Differences will be considered statistically significant at a *P* value <.05. Missing data will be handled using the multiple imputation method.

## Results

In August 2019, 6 CHWs were selected with the help of the community. From September 2019 to October 2019, 408 home caregivers with their 715 children younger than 5 years were identified in the intervention area by CHWs. Among them, 350 children younger than 5 years old and their 203 home caregivers were randomly selected and enrolled in the intervention group. In addition, CHWs identified 451 home caregivers with their 708 children younger than 5 years old. Among them, 350 children younger than 5 years old and their 225 home caregivers were randomly enrolled in the control group.

In the intervention group, 203 home caregivers were trained in November 2019 to prevent and treat malaria at home for their children younger than 5 years. Diagnostic kits (RDTs and thermometers) and treatment (artesunate-amodiaquine and paracetamol) were distributed to these home caregivers during the training. Home treatment effectively started in December 2019 and will continue until May 2020. The baseline data have already been collected and are being analyzed.

## Discussion

The goal of this study is to examine the impact of a home-based intervention strategy for the prevention and treatment of malaria in children younger than 5 years of age. Home-based management of malaria including diagnosis by RDT and treatment based on test results is a promising strategy to improve the access of remote populations to prompt and effective management of uncomplicated malaria and to decrease mortality due to malaria [10]. The home-based prevention, diagnosis, and treatment of malaria of children younger than 5 years of age is aimed at providing this group with prompt diagnosis and appropriate treatment in order to reduce severe malaria, which is the leading cause of death in children younger than 5 years.

The prevention phase of malaria consists of providing door-to-door health education to the home caregivers, which includes malaria prevention measures, such as cleaning around the house, the use of insecticide-treated mosquito nets, the use of window screens, the use of insecticides, and the closure of doors and windows before nightfall.

The management of malaria phase consists of training and providing home caregivers with malaria kits to conduct prompt diagnose and appropriate treatment of children younger than 5 years at home. Home caregivers have demonstrated excellent adherence to guidelines, potentially contributing to a decrease in malaria-related deaths in the community [10]. Our study design provides opportunities for evaluating the effectiveness of home caregiver–based prevention, diagnosis, and treatment at home for malaria in children younger than 5 years as compared to that of community treatment by CHWs. In the evaluation of each intervention’s cost-effectiveness for the primary outcomes, we hypothesize that each intervention will improve the efficacy of the home-based management of malaria.

Findings from this randomized controlled trial may contribute to resolving the challenges of severe malaria and to reducing the deaths of children younger than 5 years due to malaria. Our results may further create greater benefits for home caregivers who will be able to promptly diagnose and appropriately treat malaria in their children at home. The findings will inform public health authorities on the impact of a home-based strategy for the prevention, diagnosis, and treatment of malaria in children in Cameroon.

## Appendix

Multimedia Appendix 1 CONSORT eHealth checklist (V 1.6.1).

## Abbreviations

CDI: community-directed intervention
CHW: community health worker
HMM: home-based management of malaria
RDT: rapid diagnosis test
WHO: World Health Organization
